# Automated Extraction of Human Functional Brain Network Properties Associated with Working Memory Load through a Machine Learning-Based Feature Selection Algorithm

**DOI:** 10.1155/2018/4835676

**Published:** 2018-04-10

**Authors:** Satoru Hiwa, Shogo Obuchi, Tomoyuki Hiroyasu

**Affiliations:** Faculty of Life and Medical Sciences, Doshisha University, 1-3 Tatara Miyakodani, Kyotanabe-shi, Kyoto, Japan

## Abstract

Working memory (WM) load-dependent changes of functional connectivity networks have previously been investigated by graph theoretical analysis. However, the extraordinary number of nodes represented within the complex network of the human brain has hindered the identification of functional regions and their network properties. In this paper, we propose a novel method for automatically extracting characteristic brain regions and their graph theoretical properties that reflect load-dependent changes in functional connectivity using a support vector machine classification and genetic algorithm optimization. The proposed method classified brain states during 2- and 3-back test conditions based upon each of the three regional graph theoretical metrics (degree, clustering coefficient, and betweenness centrality) and automatically identified those brain regions that were used for classification. The experimental results demonstrated that our method achieved a >90% of classification accuracy using each of the three graph metrics, whereas the accuracy of the conventional manual approach of assigning brain regions was only 80.4%. It has been revealed that the proposed framework can extract meaningful features of a functional brain network that is associated with WM load from a large number of nodal graph theoretical metrics without prior knowledge of the neural basis of WM.

## 1. Introduction

Working memory (WM) is defined as a system for temporarily holding and manipulating information over short periods of time during complex cognitive tasks [1, 2]. It is involved in a wide range of cognitive functions, such as reading comprehension [3], reasoning [4], and problem-solving [5]. These functional cognitive abilities vary from individual to individual and reflect WM capacity [6, 7]. WM capacity not only depends on specific neural systems to maintain representation but also depends on the organization and interaction of multiple brain regions [8–10]. The *N*-back paradigm is widely used to measure WM capacity [11–13]. A meta-analysis of the neural systems involved in WM during the *N*-back task found that the (1) bilateral posterior parietal cortex, (2) bilateral premotor cortex, (3) dorsal cingulate/medial premotor cortex, (4) bilateral rostral prefrontal cortex or frontal pole, (5) bilateral dorsolateral prefrontal cortex, and (6) bilateral mid-ventrolateral prefrontal cortex are all involved in WM [14]. Multiple studies have used *N*-back tests to examine the effects of WM load variations [15, 16]. Most studies have focused on regions activated as part of the WM systems and their relationship with memory load using a univariate analysis [16–18]. However, changes in WM load have more recently been shown to modulate functional connectivity between regions [19–23]. Also, complex cognitive studies highlight the importance of conducting functional connectivity analyses over univariate analysis of functional activation [24].

Functional magnetic resonance imaging (fMRI) is frequently used to measure brain activities noninvasively. Blood oxygenation level-dependent (BOLD) time series obtained by fMRI analysis is used to determine changes in resting-state and task-related functional connectivity in a variety of subjects and conditions [25, 26], revealing a complex network of functional connections among the many regions of the brain [27, 28]. Bullmore and Sporns have shown that graph theory metrics are able to quantitatively analyze functional and structural brain networks [27, 28]. These methods can determine whether a hub node exists in specific brain states, how it changes depending on the mental state or WM load, and how multiple nodes form a module, a community of densely interconnected nodes [29].

A typical fMRI data consists of hundreds of thousands of voxels. Multiple whole-brain parcellation schemes have been developed and/or proposed, and each contains hundreds of brain regions. The challenge is to determine the characteristic regions that make up this complex network and the WM load-dependent changes of their network properties, across a vast number of nodes. Conventional studies often select regions of interests (ROIs) identified by previous studies [30]. These analyses require knowledge of the neural basis of how the task affects brain activity a priori. In this case, a data-driven approach, which heuristically extracts the characteristic brain regions and their properties from analyzing data sets without prior knowledge, should be effective. Metzak et al. [31] have demonstrated the effectiveness of using a constrained principal component analysis to detect ROIs associated with load-dependent functional networks. However, they did not address how the identified ROIs were interconnected.

Here, we propose a novel machine learning and numerical optimization method that can (1) assess various brain regions involved in a particular task (WM load in the present study); (2) use graph theoretical metrics that quantitatively represent the interconnectivity of those regions; (3) reveal which regions are involved in load-dependent functional connectivity changes; and (4) determine which metrics appropriately represent those changes. In particular, we classify brain states during high and low WM loads using a support vector machine (SVM) with regional graph metrics as feature values. A genetic algorithm (GA), one of the most robust optimization algorithms, is applied to automatically determine the combination of regional graph metrics that maximizes classification accuracy between high and low WM loads. Furthermore, we assess whether our data-driven approach can extract the characteristic regions associated with WM.

## 2. Material and Methods

### 2.1. The Extraction Method for the Brain Regions and Graph Metrics Reflecting WM Load

Graph metrics reflecting differences in WM load were extracted to investigate related changes in participating brain networks. Using each graph metric as a feature value, we classified brain states during high and low loads. In this manner, the network features of a region most important for the WM load were determined.

Although graph metrics are calculated for each brain region, not all the regions contributed to the brain states represented. As such, we chose appropriate ROIs that had feature values associated with each WM load. Therefore, feature selection was performed using numerical optimization methods to acquire the highest classification accuracy possible.

**Figure 1:**
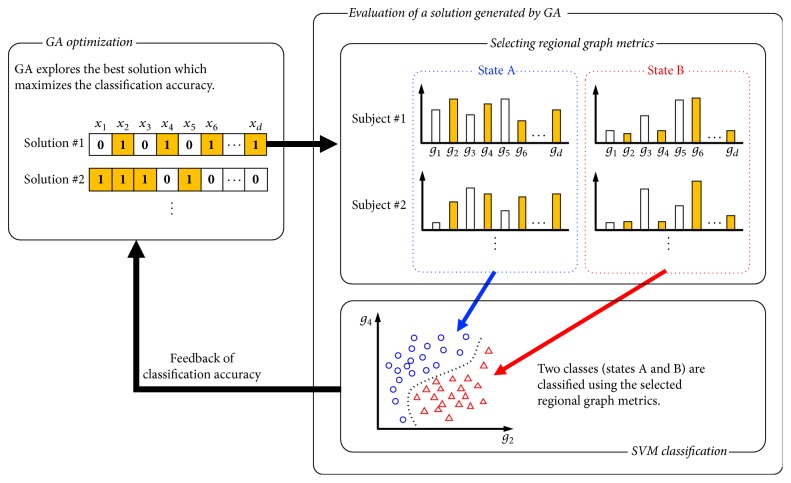
*The overview of the method*. Here *d* is the number of brain regions and *x*_*i*_ is a decision variable which determines whether the corresponding regional graph metric, *g*_*i*_ element, should be selected or deselected. States A and B correspond to the low- and high-WM load conditions, respectively.

We used an SVM to assess brain pattern classifications of WM load and a GA for feature selection. The SVM is a well-known supervised learning technique applicable for both classification and regression. It can find nonlinear solutions using the “kernel trick" in which the dataset is projected into a high-dimensional space where it can be separated linearly. The GA is an effective method for dimensionality reduction in high-dimensional classification problems because it is a heuristic, multipoint search method that can approximate optimum solutions with a relatively reasonable computation time [32, 33]. The overview of our method is illustrated in Figure 1.

In the method, the optimum combination of the regional graph metrics can be derived by solving a combinatorial optimization problem in which we aim to find the best solution that maximizes the classification accuracy of the brain state. In this optimization problem, each element decision variable *x*_*i*_ = (*i* = 1 ⋯ *d*) selects or deselects each regional graph metric *g*_*i*_  (*i* = 1 ⋯ *d*). Here, *d* is the number of brain regions, and, in this study, we set *d* = 116 because we used automated anatomical labeling (AAL) [34] for whole-brain parcellation. The objective function is the classification accuracy of the two classes (e.g., low- and high-WM load conditions) of the dataset in the feature space constructed using the SVM with the selected regional graph metrics, defined by the decision variable, **x** = (*x*_1_ ⋯ *x*_*d*_). A combination of the regional graph metrics is optimized by finding the optimum solution **x**_opt_ that maximizes the objective function *f*(**x**_opt_) = max⁡*f*(**x**). These procedures can be formulated as follows:

(1) Maximize fx,subject  to xk∈0,1.

By solving this optimization problem using numerical optimization algorithms, the characteristics of brain networks that differentiate between the two classes can be extracted automatically.

In the current study, our proposed method is applied to fMRI data during an *N*-back task to detect the regional graph metrics that reflect the WM load-dependent changes. Degree centrality, clustering coefficient, and betweenness centrality of each region were used as graph metrics to classify brain states during the 2- and 3-back tasks using an SVM. Since each graph metric was calculated for the 116 whole-brain regions for each subject, each dataset with *d* = 116 features and *n* = 30 subjects (subject information was described in Section 2.3) was trained with C-SVM implemented in LIBSVM [35] to discriminate between the 2- and 3-back tasks C-SVM which is the soft-margin SVM model which uses the cost parameter to control the cost of misclassification on the training data. Note that the classifier was created separately for each graph metric. Also, we used the radial basis function (RBF) kernel. The hyperparameters of SVM, the cost of C-SVM, and the gamma of the RBF kernel were determined by a grid search [36]. Furthermore, to acquire the average accuracy of the SVM classification, we used leave-one-subject-out cross-validation (LOSOCV).

To extract the meaningful ROIs, a GA implemented in a Deap [37] library was used. A GA used here is based on the original description of Holland's implementation [38] described in Algorithm 1. Two-point crossover was applied as the recombination operator, and the bit-flip mutation scheme which flips the value of the gene of the input individual was used as the mutation operator. Tournament selection, which selects the best individual among tournament size of four randomly chosen individuals until the number of selected individuals reaches the population size, was adopted. Moreover, the average classification accuracy via LOSOCV of the SVM model with the graph metrics chosen by the GA (forming the individual) was utilized for the fitness measure in the GA. Parameter settings of the GA are shown in Table 1.

### 2.2. Performance Evaluation of the Proposed Method

To evaluate the effectiveness of our method, we determined the combination of the regional graph metrics using a “manual" approach. In this approach, the brain regions whose graph metrics increased significantly between the 2-back to 3-back condition were selected and then classified using the SVM. These processes were applied for each graph metric: degree, clustering coefficient, and betweenness centrality. The classification accuracies of the “automatic" approach outlined above and the “manual" approach were compared to demonstrate the effectiveness of combinatorially optimizing the regional graph metrics.

### 2.3. Participants

Thirty healthy individuals (aged 20–26 years; 22 males and 8 females) with normal or corrected-to-normal vision were included in this study. This study was approved by the Ethics Committee of the Doshisha University. Written informed consent was obtained from each participant. Two participants were excluded from this study because their heads moved more than half of a voxel (2 mm) during their fMRI scans.

### 2.4. *N*-Back Task

The WM paradigm was the classical letter *N*-back task with increasing levels of memory load [17], including 0-, 2-, and 3-back versions of the task. The 0-back condition was used as a control. In the 0-back condition, participants were asked to respond when the target letter “X” appeared on the screen. As task conditions, we used the 2- and 3-back conditions. We classified brain activity during the 2- and 3-back tasks using graph metrics in each region. Typically, the 1-back condition is included to observe a load-dependent change of functional connectivity; however, we investigated whether our method could differentiate between the two higher load conditions of 2- and 3-back tasks.

**Figure 2:**
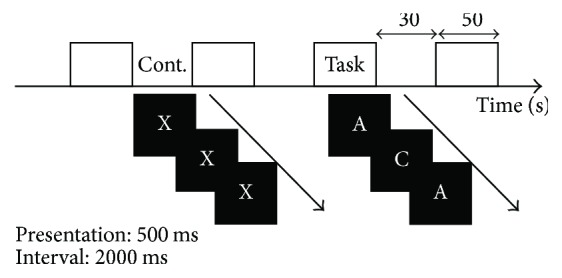
Experimental design.

During those task blocks, a pseudorandom sequence of five letters (A–E) was presented on the screen, and the participants were asked to respond if the letter was identical to the letter *N* trials before by pressing the left button; otherwise, they were instructed to press the right button. Participants performed two sessions (2- and 3-back tasks) during the MRI *N*-back scanning session. Each control condition (0-back) lasted for 30 s, and task conditions (2- or 3-back tasks) lasted for 50 s. Each block was presented four times in one session as shown in Figure 2.

Each letter remained visible for 500 ms, with an interstimulus interval of 2000 ms. Tasks were presented and synchronized with fMRI data acquisition using the Presentation software (NeuroBehavioral System), and participant responses were acquired by the fORP 932 Subject Response Package (Cambridge Research System).

### 2.5. Data Acquisition

Whole-brain imaging data were acquired with a 1.5 T MR scanner (Echelon Vega, Hitachi Corporation, Japan). For functional imaging, a gradient-echo echo-planar imaging (GE-EPI) sequence was used (TR = 2500 ms, TE = 50 ms, FA = 90°, FOV = 240 × 240 mm, matrix = 64 × 64, thickness = 6.0 mm), providing whole-brain coverage in 20 slices. For T1 anatomical imaging, an RF-spoiled steady-state gradient echo (RSSG) was used (TR = 9.4 ms, TE = 4.0 ms, FA = 8°, FOV = 256 × 256 mm, matrix = 256 × 256, thickness = 1.0 mm, and slice number = 192).

### 2.6. Preprocessing

The fMRI data were analyzed using SPM8 (Wellcome Department of Cognitive Neurology, London, UK) on MATLAB (MathWorks, Sherborn, MA). The first six images collected were discarded from the analysis in order to eliminate nonequilibrium effects of magnetization. All functional images were realigned to correct for head movements, and then anatomical images were coregistered to the mean of the functional images. Realigned functional images were normalized with the anatomical image and were smoothed using a Gaussian filter (8 mm full width-half maximum).

### 2.7. Activation Mapping

To identify activated voxels in each task condition, a boxcar reference function convolved with a hemodynamic response function was adopted for individual analysis and used in a general linear model. Using a random-effects model, we performed a paired *t*-test to examine the difference in activation between the 2- and 3-back tasks. The AAL atlas was used to determine activated brain regions. An ROI-based activation analysis was employed in order to equate it with the following functional connectivity analysis.

### 2.8. Functional Connectivity

To analyze functional connectivity, we used conn toolbox [39] on MATLAB. In addition to the preprocessing through SPM8, a band-pass filter (0.008 Hz–0.09 Hz) was applied to remove physiological and low-frequency noise. Then, we used a CompCor strategy [40] to regress out the BOLD signals from the white matter and cerebrospinal fluid and also remove subject-movement and physiological confounding effects. To calculate the correlation coefficient during task blocks (2-back and 3-back sessions), block regressors for each condition were convolved with a canonical hemodynamic response function that takes into account hemodynamic delay, and these block regressors were regressed out to remove any potential confounding effects of shared task-related responses. All scans with nonzero effects in the resulting time series were concatenated for each condition and across all sessions. We defined the brain regions as nodes and correlation coefficients as edges in applying the fMRI data to graph theoretical analysis. We chose the AAL atlas to define ROI, and Pearson's correlation coefficient was calculated between the seed time courses and those of all other regions. These correlation coefficients were converted to normally distributed scores using Fisher's transformation for group analysis. A total of 116 × 116 whole-brain, functional connectivity matrices were generated for each session across all participants.

We used only positive connections to calculate the graph theoretical metrics, rather than using negative weights, following well-established precedent [41, 42]. Graph theoretical metrics, such as degree, clustering coefficient, betweenness centrality, and modularity, were computed using the Brain Connectivity Toolbox [43]. Degree is defined as the number of links connected to a single node. In functional connectivity analysis, regions with high degrees indicate that they cooperate with many others. The clustering coefficient is the fraction of triangles around a node and is equivalent to the fraction of that node's neighbors that are neighbors of each other. Therefore, it is a measure of the tendency of nodes to cluster into strictly connected neighborhoods. Betweenness centrality is the fraction of the shortest paths within the network that contains a given node. Nodes with high values of betweenness centrality indicate the most central nodes in a graph, as they act as bridges between other nodes. Modularity subdivides a network into separate modules by maximizing the connections within each module and minimizing the number of connections between modules. Here we applied the Newman modularity algorithm [44] to the functional connectivity matrix that was averaged across all participants. After the modules were identified, the BrainNet Viewer [45] was used to visualize them.

## 3. Results

### 3.1. Behavioral Performance

Average correct answers for the 2- and 3-back tasks were 94.5% ± 5.11% and 85.84% ± 7.59%. Correct answers for the 2-back task were significantly higher than those for the 3-back task [*t*(27) = 4.88, *p* < 0.001].

### 3.2. Activation Patterns

**Figure 3:**
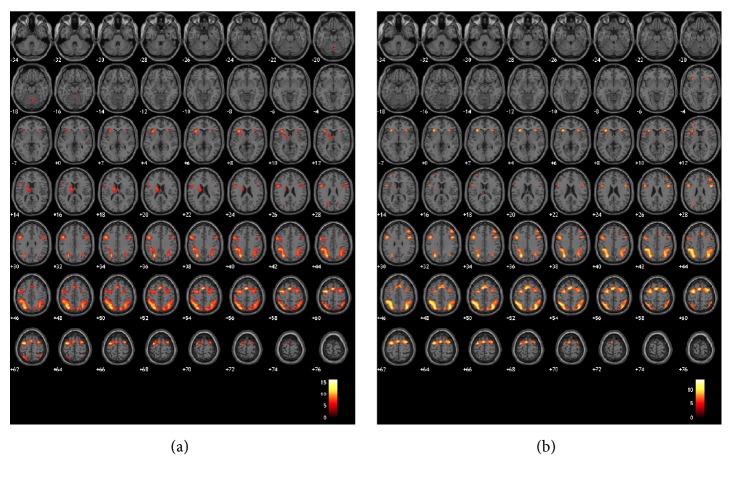
Activation pattern for the (a) 2-back task and (b) 3-back task, comparing the control and task conditions.

Figure 3 shows activated regions for the 2- and 3-back tasks based on the comparisons of task and control condition data. Significantly activated regions are shown in Table 2. However, there was no significant difference between the 2- and 3-back tasks with respect to differential brain activity (i.e., different activated regions) by random-effects theory (*p* < 0.05; corrected).

### 3.3. Functional Connectivity

**Figure 4:**
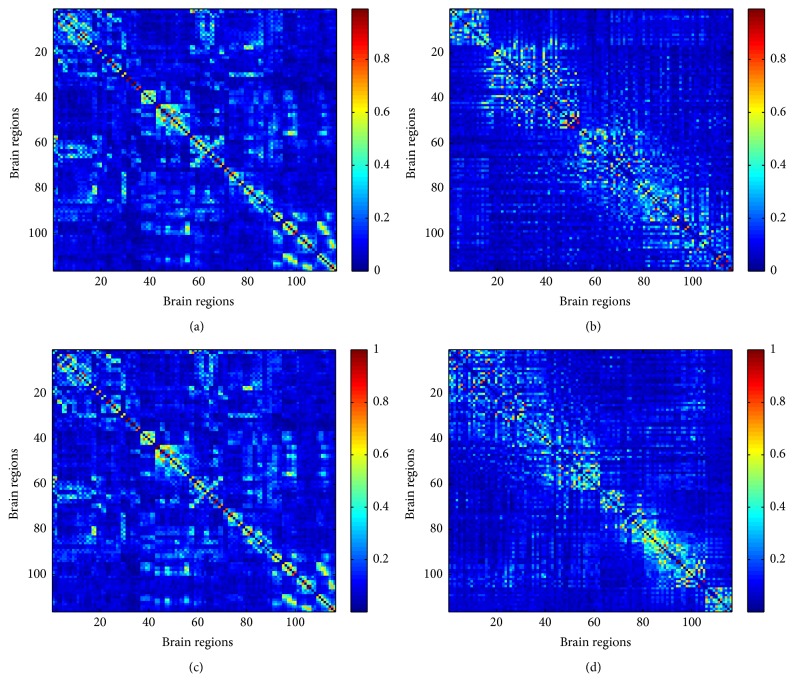
* Group-averaged connectivity matrix for the 2- and 3-back tasks*. (a) Functional connectivity for the 2-back task. (b) Functional connectivity for the 2-back task reordered using modularity. (c) Functional connectivity for the 3-back task. (d) Functional connectivity for the 3-back task reordered using modularity.

Correlation coefficients between the brain regions were calculated using BOLD fMRI signals. Average functional connectivity matrices for the 2- and 3-back tasks are shown in Figures 4(a) and 4(c). Functional connectivity matrices that were reordered by defining modularity for the 2- and 3-back tasks are shown in Figures 4(b) and 4(d).

**Figure 5:**
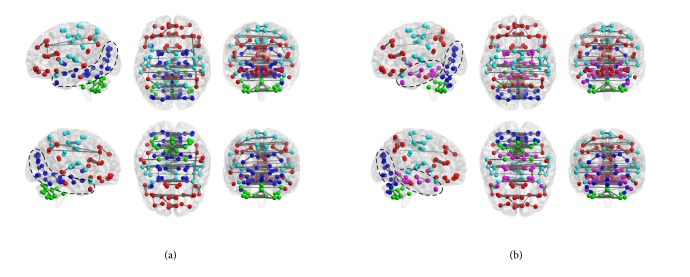
*Functional connectome*. Degrees of connectivity of the group-averaged connectome for the 2-back (a) and 3-back tasks (b) are shown. Node colors indicate distinct clusters identified using Newman algorithm implemented in Brain Connectivity Toolbox. Edges reflect correlation coefficients higher than 0.5.

Vertical and horizontal axes indicate brain regions, and each cell shows the correlation coefficient between regions. Clustered functional graphs in anatomical space are shown in Figure 5.

The number of modules for the 2- and 3-back tasks were 4 and 5, respectively, and modularity values were 0.1407 and 0.1421. Figure 5 illustrates the depth of the brain and the difference in clustering with regard to the density of connections between the occipital lobe (indicated as dashed areas). Regions which had a significantly higher graph metric value in the 3-back task than in the 2-back task are listed in Table 3 as are the classification accuracy of each graph metric, the degree, the clustering coefficient, and the betweenness centrality of these regions.

**Figure 6:**
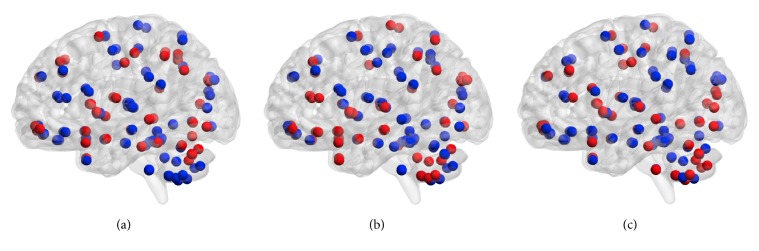
*Anatomical location of the brain regions selected by the method*. Each sphere indicates the anatomical location determined by the automated anatomical labeling (AAL) atlas. Red spheres indicate the regions selected by our method. Each panel shows regions selected using one of the graph metrics: degree (a), clustering coefficient (b), and betweenness centrality (c).

The brain regions selected automatically by our method and the classification accuracy using them are shown in Table 4. In Figure 6, the anatomical locations of the regions selected by our algorithm and which match the AAL atlas are colored red.

## 4. Discussion

The behavioral data collected during our *N*-back testing indicated that the increased WM load from the 2-back to 3-back tasks affected participants' performance. We expected that this increased WM load would be reflected in neural activation. Indeed, the brain regions activated during the 2- and 3-back tasks (shown in Table 2) are consistent with those previously reported [14]. However, there was no statistically significant difference in the specific regions activated between the 2- and 3-back tasks as analyzed using the method implemented on SPM8 (Figure 3). Despite the similarity of regions activated, the functional connectivity between the regions did differ significantly between the two task conditions.

Though both the frontal area (Frontal Mid) and parietal region (Parietal Inf) were activated under both WM tasks, functional connectivity between these regions decreased from the 2-back to the 3-back test. This result differs from other studies which report that functional connectivity increases with increasing the WM load [21–23]. The difference between our results and that of the others may be attributed to differences in the experimental paradigms used. When WM load is excessive, brain activation decreases through what is termed overflow [46]. In our experiment, several subjects may have been in this “overflow" state, causing a reduction in the detected functional connectivity.

The modules of the brain illustrated in Figure 5 indicate that the occipital lobe and deep brain are more densely connected during the 2-back task. Therefore, the functional connectivity between the occipital lobe and the deep brain has been suggested to be involved in the WM load. However, the modularity observed, which reflects the quality of clustering, was lower than the values that have been previously reported (0.2 to 0.3) in social networks. Here because modularity was less than 0.2, the precision of clustering was not sufficient. One contributing factor may have been the method of creating group-averaged functional connectivity matrices. The functional connectivity matrix used to calculate modularity excluded negative values and was obtained by averaging the correlation coefficients of all subjects. Differences between the brain networks within each subject are thought to affect the approach to and performance of each task. Therefore, simply averaging all subjects' networks may have affected the accuracy of clustering. Therefore, group analysis techniques of functional connectivity matrices should be considered in future studies.

Conversely, degree, clustering coefficient, and betweenness centrality of several identified regions were found to be significantly higher during the 3-back task than during the 2-back task. Increases in the degree between the Frontal Mid R, Sup Motor Area L, rectus R, fusiform L, and thalamus R indicated cooperation between these regions to cope with the increased WM load. The Precentral R, Frontal Inf Tri L, Temporal Pole Sup L, Temporal Mid L, Cerebellum Crus 1 L, and Cerebellum 8 R all exhibited increased betweenness centrality from the 2-back to the 3-back tasks indicating that, under increased WM load, these regions became central bridging nodes between disparate brain regions. Under increased WM load, the clustering coefficients between the Olfactory L, caudate L, Frontal Med Orb R, Cingulum Ant L and R, Lingual R, and Occipital Sup R indicated that these regions strengthened their connections. According to Osaka et al. [47], the WM load is positively correlated with the BOLD signal in the Cingulum Ant. Although we did not observe an increased BOLD signal in the Cingulum Ant from the 2-back to the 3-back tasks, the increased correlation coefficients of the Cingulum Ant suggest that it plays important roles in connecting neighboring nodes and in synchronizing activity with frontal areas during increased WM load. Because we only used significantly increased graph metrics for classification (Table 3), classification accuracy was low. This suggests that the difference between the circuits recruited during the 2- and 3-back tasks is difficult to be explained by the simple fluctuation (e.g., the increase from 2-back to 3-back) of graph metrics. Similarly, extraction of the network properties reflective of WM load-dependent change by the conventional “manual" approach may also be problematic.

We were able to classify the patterns of brain activation by performing pattern recognition of network features in 116 brain regions (Table 4). However, there are some limitations with the present approach. Future work should address several issues. First, we used the AAL atlas in this experiment; however, functional connectivity depends largely on the atlas used. Second, in segmenting the regions, the amount of information is drastically reduced because thousands of voxels are averaged into a single value making fine-grained identification of regional brain changes impossible. Novel approaches for segmenting regions and voxel-based analysis are important considerations for future studies. In particular, structural parcellations such as the AAL and Brodmann area may be inappropriate for functional connectivity analysis as anatomical boundaries do not necessarily correlate with functional ones [48]. Therefore, functional parcellations such as the Power [49, 50] and Craddock et al. [51] atlases might be more appropriate. Furthermore, we used leave-one-subject-out cross-validation for evaluating classification accuracy, but this method may include some inherent biases. Bootstrapping may avoid these issues.

Another limitation of our work is the randomness of GA optimization. In general, the GA is used to find the suboptimal solutions within the reasonable computational time, and its control parameters such as the number of initial population and iterations, crossover rate, and mutation rate significantly affect the variability of the obtained solutions. This may lead to less reproducibility of the characteristic network properties associated with the dataset. It should be further investigated how much the selected ROIs vary due to them. However, the evolutionary computation techniques, such as the GA, an evolutionary strategy, and a particle swarm optimization, have been widely used in neuroscience researches [52–54]. Moreover, Baldassarre et al. [55] tried to interpret the classifier weights of the sparse models for classifying fMRI images during visualization of pleasant and unpleasant pictures. They stated that interpretability and stability of the predictive brain maps can be affected by the model selection. Their result suggests that the reproducibility issue is not only for the evolutionary algorithms. It should be further discussed how to resolve this problem. In spite of this drawback, since it is hard to choose the seed ROIs and their network metrics for the dataset whose neural basis has not yet been investigated, in that case the proposed method will be valuable even if the obtained results would have the higher variability.

Furthermore, because we adopted a nonlinear kernel for the SVM, it made it harder to interpret the relation between the selected ROIs and the classification boundary. A possible future direction could be to evaluate the proposed method on the linear classifier.

Irrespective of these limitations, the present work developed a framework for optimizing the combination of regional graph metrics to extract meaningful ROIs and for using these graph metrics to associate WM load-dependent changes with functional connectivity networks.

## 5. Conclusions

The present work implemented a machine learning-based feature selection algorithm for extracting network properties such as functional connectivity that are associated with WM load using an *N*-back paradigm and fMRI. Our method consisted of two machine learning algorithms: (1) a GA that optimized the combination of regional graph theoretical metrics and (2) an SVM that classified two different brain states. The method first identified which brain regions should be used for classification and used three regional graph theoretical metrics, namely, degree, clustering coefficient, and betweenness centrality to classify these regions under the testing conditions.

The experimental results showed that our method could obtain more than 90% of classification accuracy using each of the three graph metrics. On the other hand, the accuracy of the manual approach that classified the regions whose graph metrics increased significantly from the 2-back to 3-back task was up to 80.36% in three graph metrics. The data indicate that our method outperforms a conventional manual approach to choosing ROI. Although, in the present study, our method was applied to the analysis of WM load-dependent functional network changes, its framework is more broadly applicable to other experimental paradigms seeking to identify, parcellate, and classify regional brain features that differentiate between two cognitive states. We are in the process of validating the method within various additional experimental scenarios.

## Figures and Tables

**Algorithm 1:**
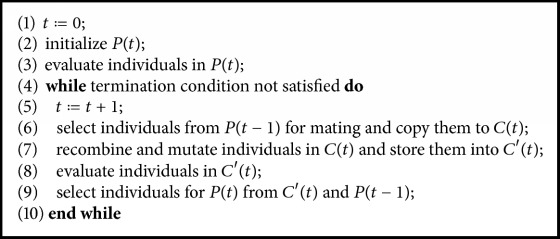
Genetic algorithm.

**Table 1:** Parameters of GA.

GA parameter	Value
Population size	100
Gene length	116
Number of generations	100
Crossover method	Two-point crossover
Crossover rate	0.7
Mutation rate	0.5
Selection method	Tournament selection (tournament size: 4)

**Table 2:** Brain regions that were significantly activated between the control and 2-back or 3-back tasks (FWE-corrected, *p* < 0.05).

Task	Region	*x*	*y*	*z*	*Z* value	Cluster size
2-back	*Precentral L*	−34	−2	60	Inf	2584
	Supp Motor Area L	−2	10	56	7.63	
	*Parietal Sup L*	−26	−70	52	7.65	2865
	Parietal Inf L	−28	−68	42	7.32	
	*Frontal Sup R*	28	0	62	6.66	718
	*Parietal Inf R*	30	−56	50	6.61	2083
	Precuneus R	12	−72	50	6.20	
	Parietal Sup R	44	−51	60	6.05	
	*Insula L*	−34	18	8	6.58	331
	Frontal Inf Tri L	−30	32	6	5.24	
	*Caudate L*	−16	−6	16	6.07	450
	Thalamus L	−14	−14	18	5.52	
	*Precentral R*	52	10	36	5.95	290
	*Insula R*	34	22	6	5.57	113
	*Vermis 6*	4	−64	−20	5.47	62
	*Caudate R*	20	−6	24	4.96	4
	*Vermis 1 2*	2	−34	−16	4.88	8
	*Frontal Mid L*	−38	50	24	4.76	1
	*Varmis 3*	−2	−28	−12	4.73	2
	*Temporal Inf L*	−48	−54	−16	4.72	1
	*Frontal Mid R*	40	34	36	4.70	2

3-back	*Parietal Inf L*	−42	−54	52	7.49	2334
	*Precentral L*	−30	−2	60	7.38	4437
	Supp Motor Area L	−8	10	52	7.13	
	*Angular R*	36	−66	48	7.30	1993
	Parietal Inf R	44	−44	54	6.12	
	*Frontal Mid R*	38	36	32	6.03	299
	*Insula R*	34	22	8	5.88	204
	*Frontal Mid L*	−30	54	12	5.58	61
	*Putamen L*	−20	0	14	5.26	43
	Caudate L	−18	0	22	4.72	
	*Thalamus L*	−16	−22	18	4.93	10
	*Pallidum L*	−18	4	0	4.77	2

**Table 3:** Brain regions in which degree, clustering coefficient, and betweenness centrality values were significantly increased in the 3-back task and the classification accuracy using each graph metric of these regions.

	Degree	Clustering coefficient	Betweenness centrality
Accuracy	80.36	73.21	60.71

Cost	5.12 × 10^2^	1.28 × 10^2^	3.13 × 10^−2^

Gamma	4.88 × 10^−4^	5.00 × 10^−2^	7.81 × 10^−3^

Region	Frontal Mid R	Olfactory L	Precentral R
	Supp Motor Area L	Caudate L	Frontal Inf Tri L
	Rectus R	Frontal Med Orb R	Temporal Pol Sup L
	Fusiform L	Cingulum Ant L	Temporal Mid L
	Thalamus R	Cingulum Ant R	Cerebellum Crus1 L
		Lingual R	Cerebellum 8 R
		Occipital Sup L	

**Table 4:** The brain regions selected automatically by our proposed method and the classification accuracy using them.

	Degree	Clustering coefficient	Betweenness centrality
Accuracy [%]	91.07	92.86	91.07

Cost	8.00	3.28 × 10^4^	3.13 × 10^−2^

Gamma	3.05 × 10^−5^	3.13 × 10^−2^	3.05 × 10^−5^

Region	Frontal Sup L	Frontal Sup L	Precentral R
	Frontal Sup R	Frontal Sup Orb R	Frontal Mid L
	Frontal Sup Orb R	Frontal Mid R	Frontal Mid R
	Frontal Mid R	Frontal Mid Orb L	Frontal Mid Orb R
	Frontal Mid Orb L	Frontal Inf Tri L	Frontal Inf Oper R
	Frontal Mid Orb R	Frontal Inf Tri R	Frontal Inf Tri L
	Rolandic Oper L	Frontal Inf Orb L	Frontal Inf Tri R
	Rolandic Oper R	Frontal Inf Orb R	Rolandic Oper R
	Supp Motor Area L	Supp Motor Area R	Supp Motor Area R
	Olfactory L	Olfactory L	Frontal Sup Medial L
	Frontal Sup Medial R	Olfactory R	Frontal Med Orb L
	Frontal Med Orb L	Frontal Med Orb R	Frontal Med Orb R
	Insula L	Rectus R	Insula L
	Cingulum Mid L	Insula L	Insula R
	Cingulum Post R	Cingulum Ant L	Cingulum Mid L
	Hippocampus R	Cingulum Ant R	Cingulum Mid R
	Amygdala L	Cingulum Mid R	Hippocampus R
	Calcarine R	Cingulum Post R	Amygdala R
	Cuneus L	Hippocampus L	Calcarine L
	Cuneus R	Hippocampus R	Calcarine R
	Lingual L	ParaHippocampal R	Lingual L
	Occipital Inf L	Amygdala L	Occipital Mid L
	Fusiform L	Calcarine R	Occipital Mid R
	Fusiform R	Cuneus L	Occipital Inf L
	Postcentral L	Cuneus R	Postcentral R
	Parietal Inf L	Lingual L	Precuneus L
	Parietal Inf R	Occipital Sup L	Paracentral Lobule R
	Angular L	Occipital Sup R	Caudate L
	Precuneus L	Occipital Inf R	Caudate R
	Precuneus R	Parietal Sup L	Pallidum L
	Caudate L	Parietal Inf L	Temporal Sup L
	Caudate R	Angular L	Temporal Pole Sup L
	Putamen R	Paracentral Lobule L	Temporal Mid L
	Pallidum L	Paracentral Lobule R	Temporal Pole Mid R
	Pallidum R	Caudate R	Temporal Inf L
	Thalamus R	Putamen L	Cerebellum Crus1 L
	Temporal Pole Sup L	Putamen R	Cerebellum Crus2 L
	Temporal Mid L	Thalamus L	Cerebellum Crus2 R
	Temporal Mid R	Thalamus R	Cerebellum 4 5 R
	Temporal Pole Mid R	Temporal Sup L	Cerebellum 6 L
	Temporal Inf L	Temporal Pole Sup L	Cerebellum 7b L
	Cerebellum Crus1 L	Temporal Pole Sup R	Cerebellum 8 R
	Cerebellum Crus1 R	Temporal Pole Mid L	Cerebellum 9 L
	Cerebellum 6 L	Temporal Pole Mid R	Cerebellum 9 R
	Vermis 6	Temporal Inf R	Cerebellum 10 L
	Vermis 7	Cerebellum 7b L	Vermis 1 2
	Vermis 8	Cerebellum 8 L	Vermis 4 5
		Cerebellum 9 L	Vermis 8
		Vermis 7	
		Vermis 8	
		Vermis 9	
		Vermis 10	
